# Dynamics and Outcome of Macrophage Interaction Between *Salmonella* Gallinarum, *Salmonella* Typhimurium, and *Salmonella* Dublin and Macrophages From Chicken and Cattle

**DOI:** 10.3389/fcimb.2019.00420

**Published:** 2020-01-10

**Authors:** Kaisong Huang, Ana Herrero Fresno, Søren Skov, John Elmerdahl Olsen

**Affiliations:** Department of Veterinary and Animal Sciences, Faculty of Health and Medical Sciences, University of Copenhagen, Copenhagen, Denmark

**Keywords:** *S*. Typhimurium, *S*. Gallinarum, *S*. Dublin, macrophage, host specificity

## Abstract

*Salmonella* Gallinarum only infects avian species, where it causes a severe systemic infection in birds of all ages. It is generally accepted that interaction with phagocytic cells plays an important role in the development of systemic, host-specific *Salmonella* infections. The current study detailed the interaction of *S*. Gallinarum with macrophages derived from chicken (HD11) and cattle (Bomac) compared to interaction of the broad host range serovar, *Salmonella* Typhimurium and the cattle adapted serovar *Salmonella* Dublin. Results showed a weaker invading ability of *S*. Gallinarum in both kinds of macrophages, regardless whether the bacteria were opsonized or not before infections. However, opsonization of *S*. Gallinarum by chicken serum increased its intracellular survival rate in chicken macrophages. No significant induction of nitrogen oxide was observed in the infected HD11 cells within the first 6 h, and levels of reactive oxygen species (ROS) were similar among the three serovars. *S*. Gallinarum infection was associated with low cell deaths in both chicken and cattle macrophages, whereas *S*. Dublin only induced a comparable high level of cell death in chicken macrophages, but not in macrophages of its preferred host species (Bomac) compared to host generalist *S*. Typhimurium. *S*. Gallinarum-infected HD11 macrophages exhibited low induction of pro-inflammation genes [interleukin (IL)1β, CXCLi1, and CXCLi2] compared to the two other serovars, and contrary to the other serovars, it did not induce significant downregulation of Toll-like receptor (TLR)2, TLR4, and TLR5. In *in vivo* infection of 1-week-old chicken, a significant upregulation of the TLR4 and TLR5 genes in the spleen was observed in *S*. Gallinarum-infected chickens, but not in *S*. Typhimurium-infected chicken at 5 days post-infections. Taken together, results show that *S*. Gallinarum infection of macrophages was characterized by low uptake and low cytotoxicity, possibly allowing long-term persistence in the intracellular environment, and it caused a low induction of pro-inflammatory responses.

## Introduction

*Salmonella* of the Enterobacteriaceae family includes two species; *Salmonella enterica* and *Salmonella bongori*. Based on the somatic O, flagella H, and Vi-antigen variations, it is divided into more than 2,600 serovars, with around 1,600 serovars belonging to *S. enterica* subspecies *enterica* (Grimont Pad, 2007). *Salmonella* can affect a wide range of warm-blooded animals including humans and livestock (Baumler et al., 1998; Uzzau et al., 2000). Most serovars, such as *Salmonella* Typhimurium and *Salmonella* Enteritidis, are host generalists and cause disease in a wide range of species. These serovars are associated with food-borne infection estimated to affect >90 million people and cause 155,000 deaths annually in the world (Majowicz et al., 2010; Gal-Mor et al., 2014). Certain *Salmonella* serovars, however, have evolved to only cause infections in one species exclusively or in a few species, but with one preferred host species. These two groups are termed host-specific and host-adapted serovars, respectively (Uzzau et al., 2000). The host-specific group encompasses important pathogens, such as *S*. Typhi and *S*. Gallinarum, which cause typhoid fever in humans and fowl typhoid in avian birds (Shivaprasad, 2000; Kwon et al., 2010; Buckle et al., 2012). *S*. Dublin is a typical host-adapted serovar, mainly causing infections in cattle, but also sporadically reported from human infections (Nielsen, 2013; Harvey et al., 2017).

Little is known about the mechanisms that contribute to *salmonella* host specificity, although genomic differences particularly pseudogenes variations between broad host range and narrow host range serovars have been reported (Thomson et al., 2008; Wigley, 2016). Systemic infection with survival inside phagocytic cells is a hallmark of the host-specific serovars (Haraga et al., 2008; Fabrega and Vila, 2013). After passing the intestine epithelial barrier, they are engulfed by host phagocytic cells, including dendritic cells and macrophages (Galan and Curtiss, 1989; Haraga et al., 2008; Bruno et al., 2009). This induces the delivery of an array of effector molecules *via* the *Salmonella* Pathogenicity Island 2 (SPI-2) encoded type three secretion system to facilitate the intracellular survival by manipulating the unfavorable intracellular environments (Figueira and Holden, 2012; Fabrega and Vila, 2013; Srikumar et al., 2015). It has been proposed that this intracellular niche confers a safe haven where the bacteria cannot be affected by components of the humoral host defense system (Jones et al., 2001; Haraga et al., 2008).

To identify characteristics of the host-specific interaction between *S*. Gallinarum and macrophages from its preferred host, the current study compared the interplay of strains of the host generalist *S*. Typhimurium, the host-specific *S*. Gallinarum, and the host-adapted *S*. Dublin with cultured chicken and bovine macrophages, including characterization of immune gene induction caused by infection with these serovars. In addition, the *in vivo* immune response in chicken after *S*. Gallinarum and *S*. Typhimurium infection was analyzed and compared as well.

## Materials and Methods

### Strains and Culture Conditions

A representative strain of each serovar was used. The three strains, *S*. Typhimurium 4/74 (Wallis et al., 1995), *S*. Gallinarum G9 (Barrow et al., 1994), and *S*. Dublin 3246 (Bolton et al., 1999) were routinely cultured in Luria Bertani (LB) medium (Oxoid, Denmark) at 37°C with aeration and shaking at 200 rpm/min or grown in LB agar plates. For cytotoxicity assays, two more strains from each serovar, namely, *S*. Typhimurium ATCC 14028, *S*. Typhimurium D23580, *S*. Gallinarum 1904, *S*. Gallinarum 1908, *S*. Dublin 2229, and *S*. Dublin 228.89, were also included. For use as inoculum for macrophage infection, strains were grown to OD_600_ 0.6–0.8 in LB medium and then diluted to OD_600_ 0.2, corresponding to ~2 × 10^8^ colony-forming units (CFU)/ml, and eventually cultured on LB agar plates to determine the real infection dose.

### Opsonization of the Bacteria

Opsonization of bacteria was performed as previously reported with some modifications (Poermadjaja and Frost, 2000). In brief, bacteria from log phase were collected and further incubated with 20% chicken serum (Sigma, Denmark) or fetal bovine serum (Gibco, Denmark) in DPBS (Gibco, Denmark) buffer (v/v) for 45 min at 37°C with gentle shaking. Following incubation, bacteria were washed three times with DPBS buffer and then resuspended in RPMI-1640 medium (Gibco, Denmark). Subsequently, the bacterial suspensions were adjusted to the required OD/concentration for the infection assays.

### Macrophage Cell Infections

Macrophage infections were performed as previously reported with minor modifications (Setta et al., 2012; Herrero-Fresno et al., 2014). Briefly, the chicken macrophages, HD11, originally derived from chicken bone marrow cells (in store) and the cattle macrophages Bomac (in store) were grown in RPMI-1640 medium with 10% fetal bovine serum after recovering from liquid nitrogen. For infection assays, the cells culturing in the flasks between 5 and 15 passages were collected and then seeded in 24-well tissue culture plates (HD11 seeding 4 × 10^5^ and Bomac seeding 1 × 10^5^) for overnight growth in a humidified incubator with 5% CO_2_ as previously described (Setta et al., 2012; Herrero-Fresno et al., 2014). Before use for invasion assays, it was ensured by microscopy that cultures reached over 80% confluence. For preparation of the inoculum, the strains were prepared as described above and finally suspended in RPMI-1640 medium. Bacteria were added to macrophages with a multiplicity of infection (MOI) of 5:1, further incubated for 30 min, and then washed with DPBS buffer three times. In addition, experiments with Bomac using an MOI of 100:1 and 1 h incubation before addition of gentamycin were included specifically for measurement of cytotoxicity, nitric oxide (NO) production, and immune gene expression assays. For killing of the extracellular bacteria, the macrophages were then incubated with fresh RPMI-1640 medium containing 100 μg/ml gentamycin and 10% FBS for 1 h. To monitor the uptake and intracellular *Salmonella* survival and replication, the macrophages were lysed with 0.9% NaCl containing 0.1% triton X-100 after being washed three times with DPBS. The lysates were plated on LB agar plates to determine the amount of live intracellular bacteria. Counting was performed at T0, T2, and T4 h after adding 100 ug/ml gentamycin. Fold net replications at T4 were estimated relative to CFUs observed at T0.

### Macrophage Cytotoxicity Assays

Cell death due to infection with *Salmonella* was determined by measuring the released cytosolic lactate dehydrogenase (LDH) in the cell supernatant as previously reported (Meunier et al., 2014). Briefly, macrophages were infected as described above. Cell supernatants from both infected and uninfected macrophages (control) were collected at 12 h post-infection. The released LDH amounts were measured using a colorimetric Cytotox 96 assay (Promega, Denmark). Cytotoxicity was calculated using the following formula in which the maximum release was the value obtained from cell lysate:

$$\begin{array}{l} \%\text{Cytotoxicity}=\left(\text{Infected cell release}-\text{ uninfected cell release}\right)\\ \;/\left(\text{maximum release }-\text{ uninfected cell release}\right)\times 100. \end{array}$$

### Quantification of NO Production

Quantification of NO produced by macrophages in response to the *Salmonella* infection was performed using the Griess reagent (Sigma-Aldrich) as previously described (Meunier et al., 2014). Measurements were conducted at 6, 12, and 24 h post-infection, using aliquots of 500 μl cell supernatant, which were mixed with the same volume of Griess reagent. After 15 min incubation at room temperature, the NO concentration was determined by measuring the absorbance at 540 nm. Sodium nitrite (Sigma-Aldrich) dilutions were used as a standard to generate a calibration curve.

### Production of Reactive Oxygen Species (ROS)

ROS induced by *Salmonella* infection in macrophages was measured using 2′,7′-dichlorofluorescein diacetate (Sigma) as substrate as previously reported (Setta et al., 2012). Briefly, ~10^6^ macrophage cells in 1 ml RPMI-1640 medium, supplemented with 10 μg of 2′,7′-dichlorofluorescein diacetate, were infected with strains of *Salmonella* with an MOI of 5:1. After 1 h of incubation at 37°C with 5% CO_2_, aliquots of 150 μl cell culture were transferred into 96-well plates, and fluorescence intensity was measured at 485/520 nm using a Fluostar Omega micro-plate reader (BMG LEBTECH).

### Measurement of Cytokine Secretion and Toll-Like Receptor Expression

Chicken and cattle macrophages were infected with opsonized bacteria as described above. At 6 h post-infection, infected and uninfected cells were collected, and total RNA was isolated by using the RNeasy Mini kit (Qiagen, Denmark) according to the manufacturer's instructions. RNA degradation and purity were assessed by agarose gel electrophoresis and Nanodrop analysis, respectively. After being treated with DNase following the manufacturer's instructions (Promega, Denmark), RNA was transcribed into cDNA using the GoScript™ Reverse transcription system (Promega, Denmark) following the manufacturer's instructions. Transcripts were then assessed by qPCR analysis, which was performed using the SYBR FastStart Essential DNA Green master kit (Roche, Denmark) on the Roche LightCycler 96 Real-Time PCR machine. The housekeeping gene *GAPDH* (glyceraldehyde-3-phosphate dehydrogenase) was used as an internal control. The relative gene expression of cytokines [interleukin (IL)1β, IL6, IL10, LITAF, interferon (IFN)-γ, IL18, CXCLi1, and CXCli2] and Toll-like receptors (TLR1, TLR2, TLR3, TLR4, TLR5, TLR7, TLR15, and TLR21) (primer sequences listed in Supplementary Tables 1, 2) was calculated and analyzed using the 2^−ΔΔ*Ct*^ method (Livak and Schmittgen, 2001).

### *In vivo* Immune Responses to Infection With *S*. Typhimurium and *S*. Gallinarum

One-week old chickens were challenged with 2 × 10^9^ CFUs of bacteria orally as previously described (Schroll et al., 2014). The chickens were killed at 5 days post-infection by cervical dislocation, and the spleens were collected for determining the bacteria burden and related immune gene expression. For spleen *Salmonella* load, 1 g of spleen was homogenized in 0.9% sodium chloride, and 10-fold dilutions were plated on XLD agar plates. For gene expression analysis, spleen samples were homogenized in RLT buffer (RNeasy Mini kit, Qiagen) and then subjected to RNA extraction as described above. Expression of cytokine-genes [IL6, IL18, IL10, CXCLi1, and tumor necrosis factor (TNF-α)] and Toll-like receptors (TLR2, TLR4, and TLR5) was then determined by RT-PCR as described above.

### Statistical Analysis

Unless otherwise stated, data analyses were performed by one-way ANOVA or two-way ANOVA analysis with Tukey's multiple comparisons posttest using the GraphPad Prism software. A difference was considered statistically significant when the *P*-value < 0.05.

### Ethical Statement

Chicken infection experiments were conducted with permission to the senior author from the Danish Animal Expectorate (approval no. 2016-15-0201-00870).

## Results

### Uptake and Survival of *S*. Typhimurium, *S*. Gallinarum, and *S*. Dublin in Avian Macrophages HD11 and Cattle Macrophages Bomac

**Figure 1:**
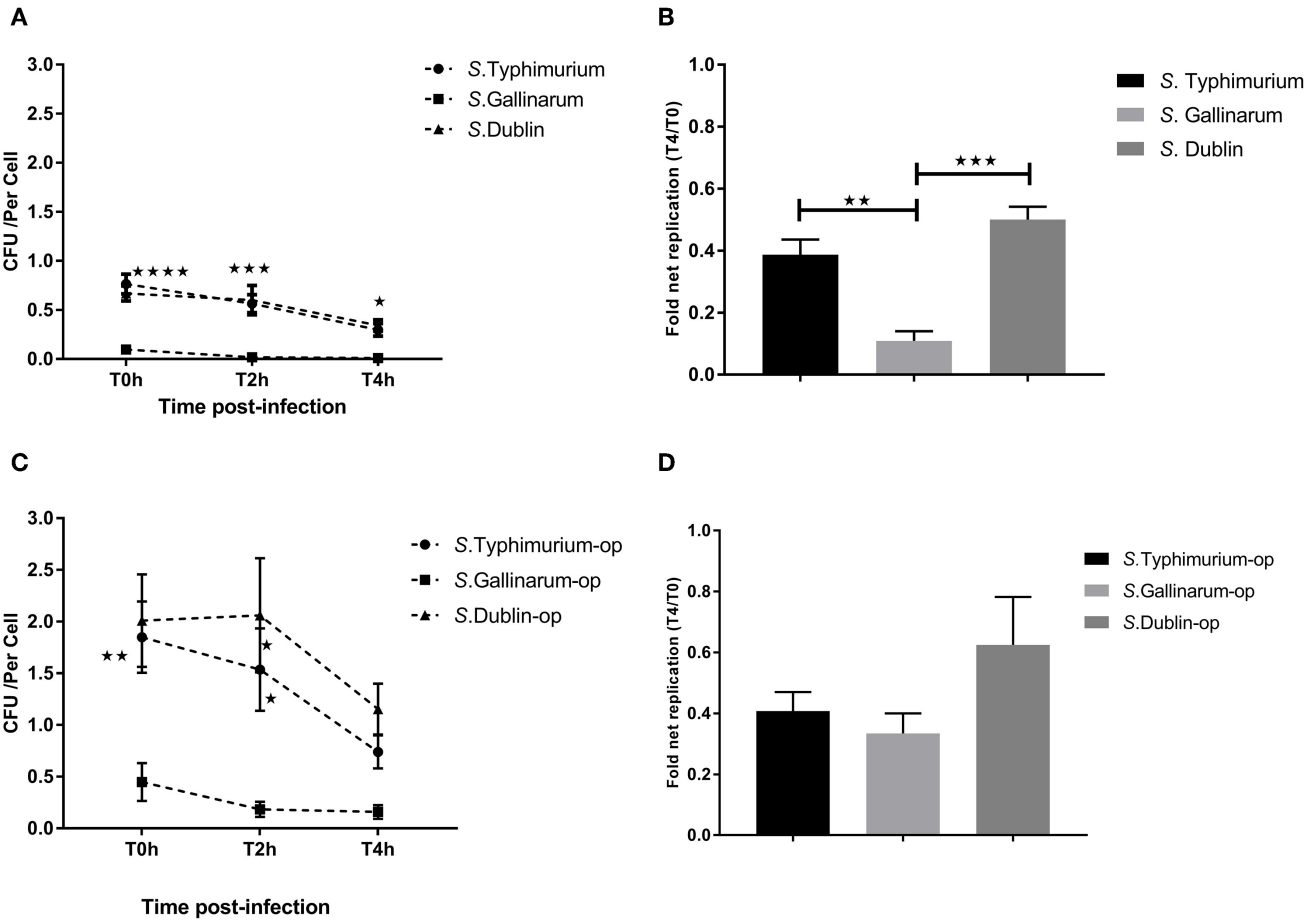
Uptake and intracellular survival of *Salmonella* strains in chicken-derived HD11 macrophages. Chicken macrophages were incubated with strains of *Salmonella* for 30 min at a multiplicity of infection (MOI) of 5:1. Cells were lysed for determination of viable intracellular bacteria at 0, 2, and 4 h after this incubation period (defining T0h, T2h, and T4h, respectively). **(A,C)** show the intracellular colony-forming units (CFU) of the strains of the three *Salmonella* serovars without **(A)** and with **(C)** opsonization before infection. **(B,D)** indicate the fold net replication without **(B)** and with **(D)** opsonization. Asterisks indicate significance between the *S*. Typhimurium or *S*. Dublin infection group with the *S*. Gallinarum group (*P < 0.05, **P < 0.01, ***P < 0.001, ****P < 0.0001). There were no significant differences between *S*. Typhimurium and *S*. Dublin.

**Figure 2:**
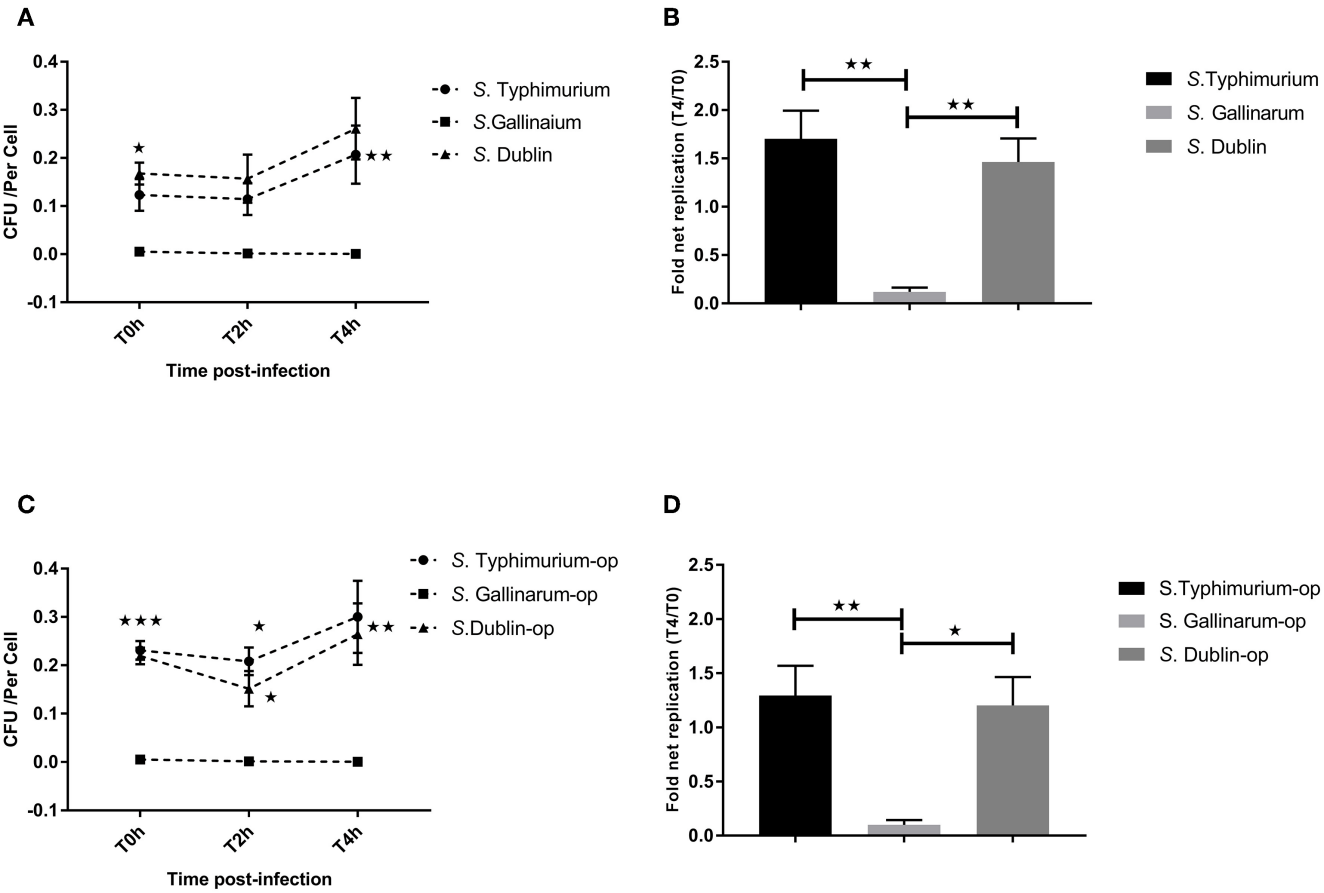
Uptake and intracellular survival of *Salmonella* strains in cattle-derived macrophages (Bomac). Bovine-derived macrophages Bomac were infected by strains of *Salmonella* at a ratio of 5:1 (incubating for 30 min). Cells were lysed for determination of viable intracellular bacteria at 0, 2, and 4 h after this incubation period (defining T0h, T2h, and T4h, respectively). **(A,C)** show the intracellular survive curve of strains of three *Salmonella* serovars without **(A)** and with **(C)** opsonization before infections. **(B,D)** show the intracellular net replication after infection without **(B)** or with **(D)** opsonization. Asterisks indicate significance between the *S*. Typhimurium or *S*. Dublin infection group with the *S*. Gallinarum group (*P < 0.05, **P < 0.01, ***P < 0.001). There were no significant differences between *S*. Typhimurium and *S*. Dublin.

Our primary aim was to identify differences between interactions of the host-specific serovar *S*. Gallinarum with macrophages of its preferred host, the chicken, compared to how the non-host-specific serotype *S*. Typhimurium and the cattle host-adapted serovars *S*. Dublin interacted with the same macrophages. A significantly higher amount of viable intracellular *S*. Typhimurium and *S*. Dublin was detected at 0, 2, and 4 h post-infection compared to *S*. Gallinarum in both HD11 macrophages and Bomac, irrespective of whether the bacteria were opsonized or not before infections (Figures 1, 2). Opsonization dramatically increased the uptake of *S*. Typhimurium and *S*. Dublin by HD11 macrophages, whereas no statistically significant increase was observed for *S*. Gallinarum infections (Figures 1A,C). All three *Salmonella* serovars were gradually killed by macrophages during the first 4 h post-infection in HD11. Interestingly, *S*. Typhimurium and *S*. Dublin showed a significant higher survival rate (T4/T0) compared to that of *S*. Gallinarum during chicken macrophage HD11 infections when the strains were not opsonized, whereas the opsonization effect increased the survival rate of *S*. Gallinarum, reaching a comparable level with that of *S*. Typhimurium and *S*. Dublin (Figures 1B,D). Uptake of *S*. Gallinarum in Bomac cells was very low, and no change in CFU over time was observed. In contrast, an increase in the number of intracellular *S*. Typhimurium and *S*. Dublin was observed over time, corresponding to net replication (Figure 2).

### Cytotoxicity Toward Macrophages HD11 and Bomac

**Figure 3:**
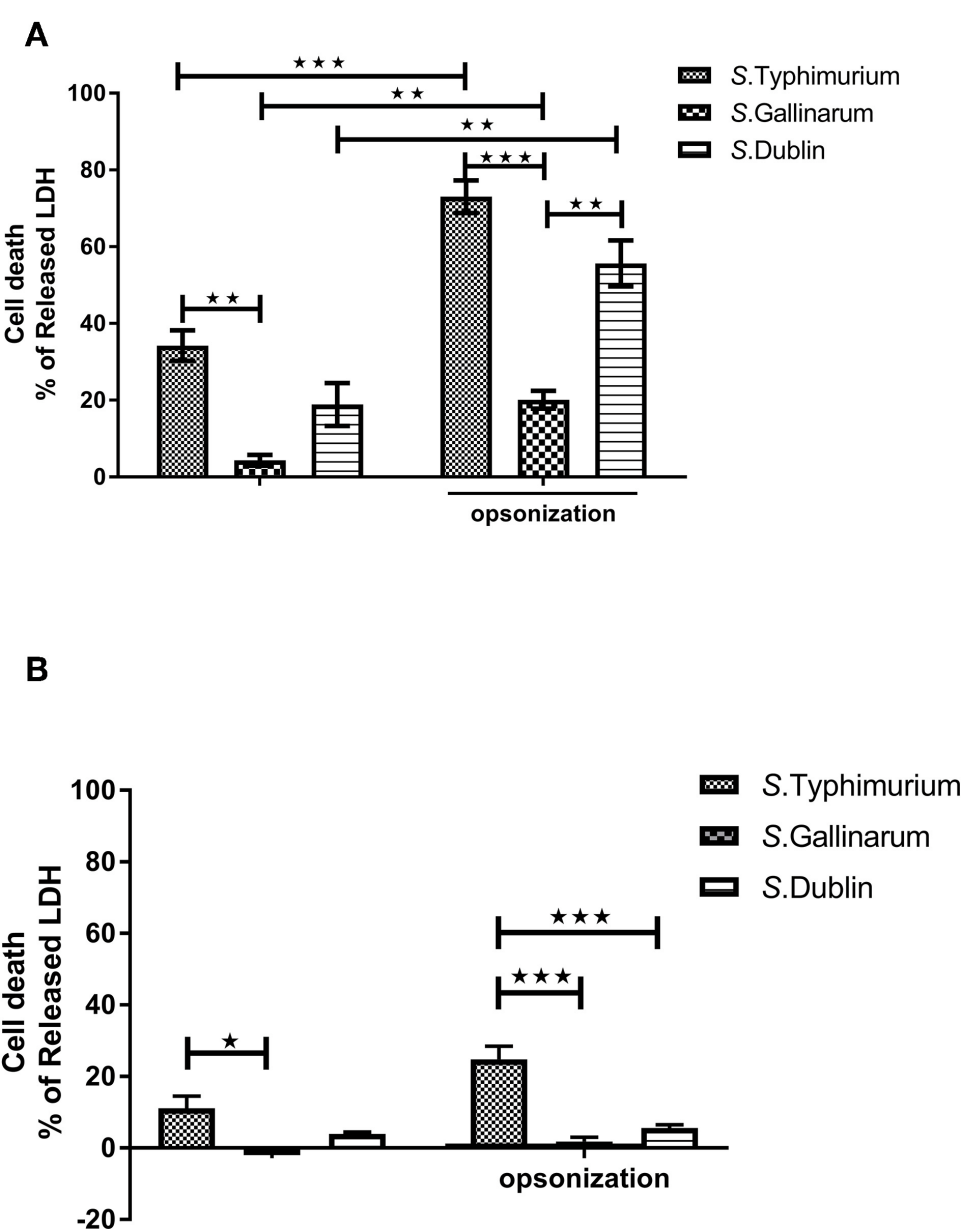
Cytotoxicity of *S*. Typhimurium 4/74, *S*. Gallinarum G9, and *S*. Dublin 3246 toward chicken-derived HD11 macrophages and bovine-derived macrophages (Bomac). The cytotoxicity was determined by measuring lactate dehydrogenase (LDH) in the cell supernatant at 12 h post-infection. HD11 macrophages were infected with a multiplicity of infection (MOI) of 5:1 **(A)**, and Bomac were infected with an MOI of 100:1 **(B)**. Asterisks indicate significance between groups (*P < 0.0 5, **P < 0.01, ***P < 0.001).

*Salmonella* has been reported to induce a high level of cell death in both epithelial and phagocytic cells after infections (Santos et al., 2001; Cardenal-Munoz et al., 2014). It is not known whether there are serovar-specific differences in this trait depending on the normal host association of the serovar, and we set out to determine whether cytotoxicity was correlated to the host specificity/host adaptation of the serovar. Both *S*. Typhimurium and *S*. Dublin infections induced a high level of cytotoxicity to chicken HD11 macrophages, whereas *S*. Gallinarum interestingly showed a low level of cytotoxicity, suggesting that host specificity could be associated with a low level of cytotoxicity (Figure 3A). A significant increase of cell death was observed for all three serovars when bacteria were opsonized in comparison with infection with non-opsonized bacteria (Figure 3A). When the same MOI and incubation times were used as for HD11 cells, no obvious cell death was detected in Bomac (data not shown). When the MOI was increased to 100:1 and incubation time to 1 h, cytotoxicity was observed, and *S*. Typhimurium induced a significantly higher cell death than *S*. Gallinarum and *S*. Dublin particularly when the bacteria were opsonized; these two serovars did not differ significantly in the levels of cytotoxicity (Figure 3B).

### NO and ROS Production by Macrophages HD11 and Bomac in Response to *Salmonella* Infection

**Figure 4:**
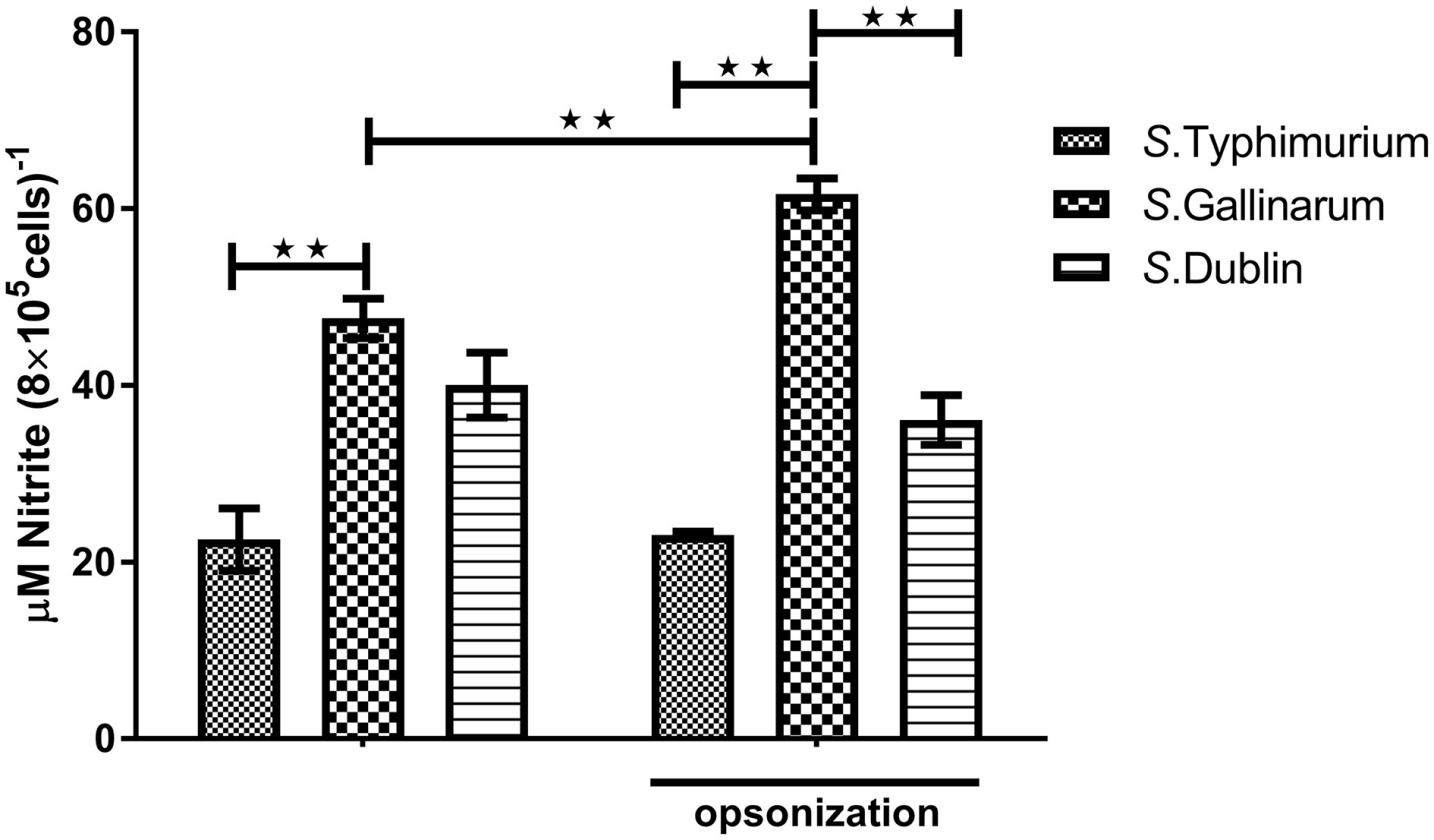
Nitric oxide (NO) concentrations in the supernatants of HD11 macrophages at 24 h post-infection with non-opsonized and opsonized strains of *S*. Typhimurium 4/74, *S*. Gallinarum G9, and *S*. Dublin 3246. The infections were performed at a multiplicity of infection (MOI) of 5:1. Asterisks indicate significance between the groups (**P < 0.01).

**Figure 5:**
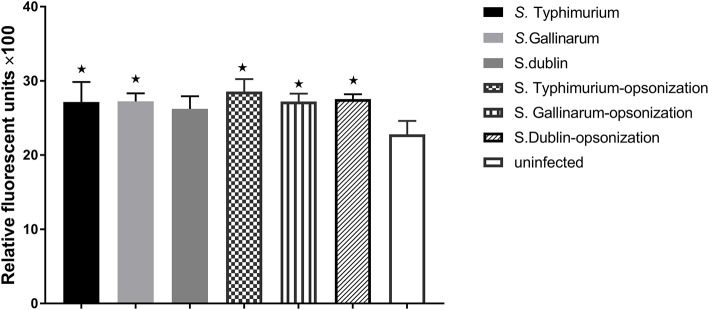
Production of reactive oxygen species (ROS) in infected HD11 macrophages 1 h post-challenge with non-opsonized and opsonized strains of *S*. Typhimurium 4/74, *S*. Gallinarum G9, and *S*. Dublin 3246. Bacteria were added to macrophages at a ratio of 5:1. Asterisks indicate significance between the infected cells and uninfected groups (*P < 0.05).

Production of NO and other ROS constitutes a significant defense against intracellular bacteria. Because we had shown above that there were significant differences on how strains of different serovars interacted with macrophages from hens and bovines, we wanted to see whether this was related to differences in activation of these defenses. However, no significant NO production was detected from infected macrophages at early time points (6 and 12 h post-infection; data not shown), and not until 24 h, did we obtain a clear readout by the method used. At this time point, significantly higher concentration of NO was observed in *S*. Gallinarum-infected cells compared to *S*. Typhimurium- and *S*. Dublin-infected cells, particularly when the strains were opsonized by chicken serum before infections (Figure 4). Reactive oxygen species, on the other hand, was induced and significantly above the level in uninfected cells already 1 h post-infection, regardless of the serovars, and regardless of whether they were opsonized prior to infection (Figure 5). No significant production of NO was detected in Bomac (MOI 5:1 and 100:1) at 24 h post-infection (data not shown).

### Immune Response During *Salmonella* Infections in Macrophages

**Figure 6:**
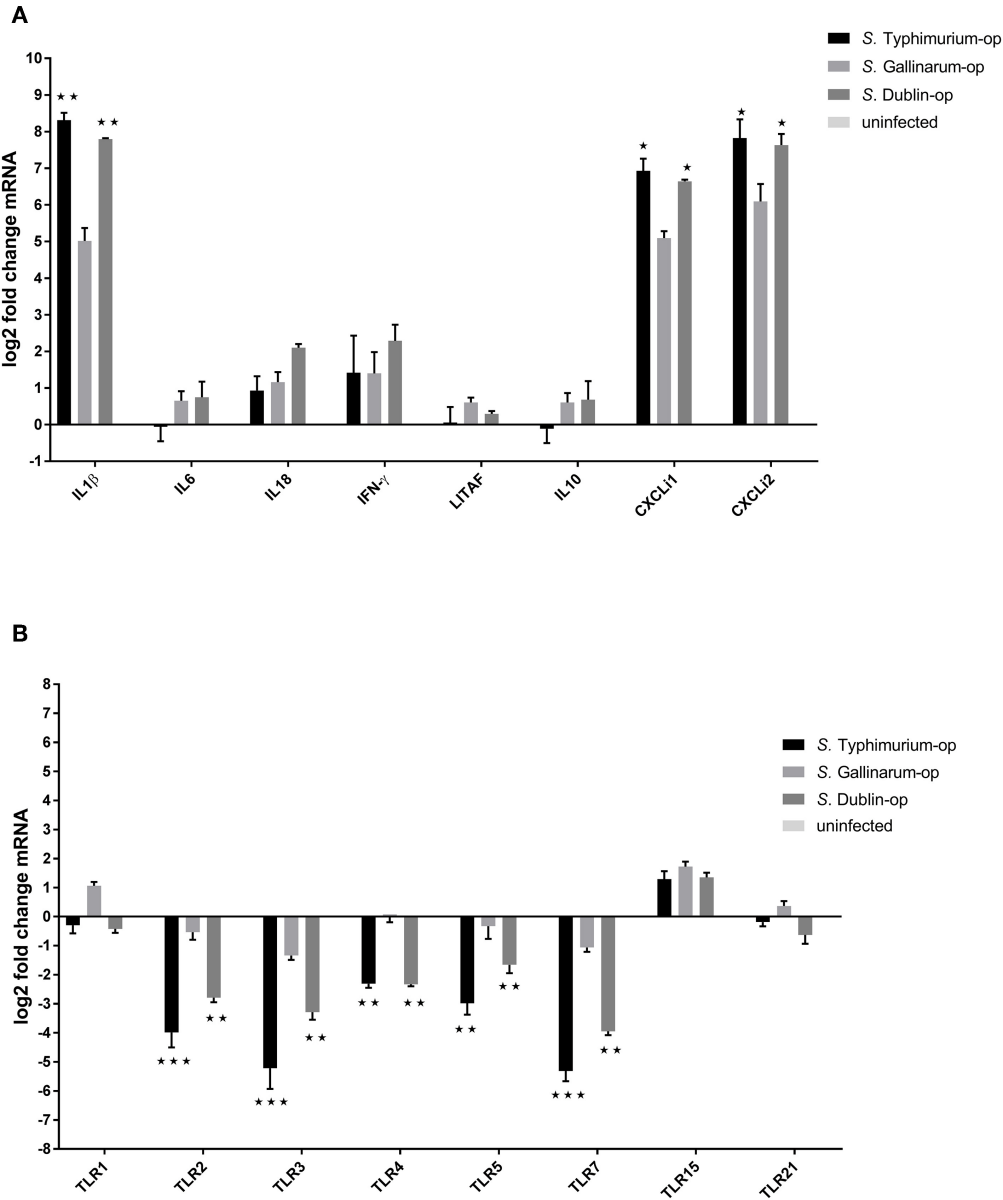
Expression of cytokines **(A)** and Toll-like receptors (TLRs) **(B)** in HD11 macrophages 6 h post-challenge with opsonized bacteria. Chicken macrophages were infected by *Salmonella* with a multiplicity of infection (MOI) of 5:1. Expression was determined by RT-PCR. Pro- and anti-inflammation cytokines, related pivotal chemokines, and innate immune receptors were examined. The uninfected cells were used as control group for calculating the fold change of differential gene expressions in the infected cells. Asterisks indicate significance between the *S*. Typhimurium or *S*. Dublin infection group and the *S*. Gallinarum group (*P < 0.05, **P < 0.01, ***P < 0.001).

It is known that host-specific and broad host range serovars differ with respect to induction of inflammation response in the host (Tsolis et al., 2008; Chappell et al., 2009), and also that the near relative of *S*. Gallinarum, the biovar Pullorum, induces a Th2 response with induction of IL4 in the chicken (Tang et al., 2018). TLR activation is known to be involved in this (Zhan et al., 2015; Johnston and Corr, 2016). We wished to determine whether the serovar differences observed above were also expressed at the level induction of pro-inflammation cytokines, chemokines, and innate immune TLRs in macrophages after infection with the three different *Salmonella* serovars, and how the three serovars differed in induction. As uptake and survival experiments had shown that opsonization resulted in uptake of more bacteria, we performed these experiments with opsonized bacteria. The results showed that the three *Salmonella* serovars induced expression of pro-inflammation cytokines IL1β, interferon-γ, and chemokine CXCLi1 and CXCLi2 in HD11 macrophages at early stages. Infection with *S*. Typhimurium triggered the highest expression of IL1β (317-fold compared to uninfected cells), CXCLi1 (121-fold), and CXCLi2 (225-fold) of the three different serovars, in particular, substantially higher than *S*. Gallinarum infection (Figure 6A). In contrast, no significant induction of expression of IL6, LITAF, and cytokine IL10 was detected after infection with the different *Salmonella* strains. A significantly increased expression of the Th1 response typical cytokine IFN-γ in HD11 was observed in all these serovar infections compared to uninfected group, whereas there was no statistically significant difference of expression level of IFN-γ between these three different serovars. The cytokine IL18, which drives the Th1 response, was only found to be statistically significantly upregulated in the *S*. Dublin infection group (4.1-fold).

Infection with *S*. Typhimurium and *S*. Dublin led to a significant downregulation of TLR2, TLR3, TLR4, TLR5, and TLR7 in chicken macrophages HD11, whereas only a slight downregulation of TLR3 and TLR7 genes was observed in the *S*. Gallinarum infection group (Figure 6B). In addition, TLR15, which is a chicken unique TLR (Higgs et al., 2006), was found to be slightly upregulated in all three groups, whereas no changes of the expression of TLR21, another chicken-specific TLR (Brownlie et al., 2009), were detected in any of the groups (Figure 6B). Expressions of cytokines IL1β, IL6, IL8, TNF-α, and IL18 and TLRs TLR2, TLR4, and TLR5 were explored in infected cattle macrophages as well. No statistically significant changes were detected for any of these immune genes compared to uninfected cells in both low (5:1) and high (100:1) multiplicity (data not shown).

### Expression of Immune Genes in the Spleen of *S*. Typhimurium- and *S*. Gallinarum- Infected Chickens

**Figure 7:**
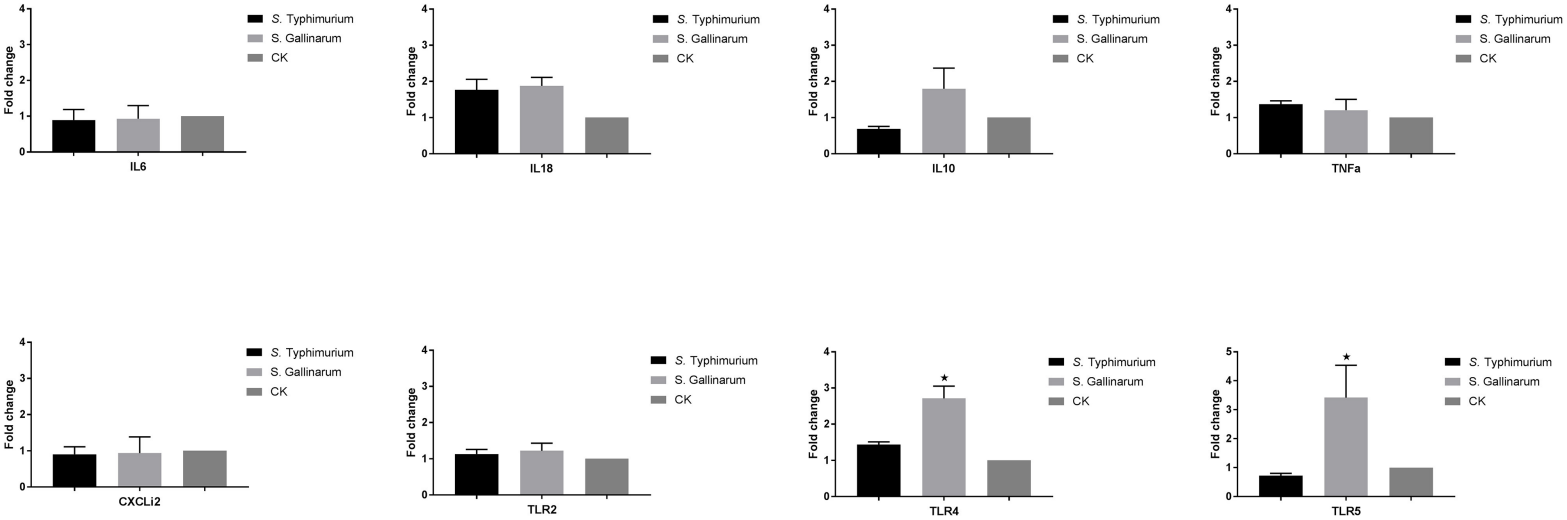
Expression of crucial immune genes of different T helper cell response, inflammation cytokines, chemokines, and innate immune receptors in the infected chicken spleen. One-week-old chicks were orally infected by *S*. Typhimurium and *S*. Gallinarum, and the spleen samples were collected for analysis at 5 days post-infection. Expression was measured by RT-PCR approach, and the uninfected chickens were used as control group (defined as CK). Asterisks indicate significance between the infected and uninfected groups (*P < 0.05).

Because we had observed differences in expression of immune genes of HD11 macrophages above, we wanted to see whether this was reflected at the systemic level of the infected hen or whether it was a particular trait of the infected macrophage. To do so, we performed a challenge experiment and determined the expression of selected immune genes in spleen cells 5 days post-infection. CFU from spleens can be seen from Supplementary Figure 1. No statistically significant difference was observed in the expression of IL6, IL10, IL18, TNF-α, CXCLi2, and TLR2 genes between *S*. Typhimurium and *S*. Gallinarum in the spleen of infected chickens and uninfected chickens. A slight but statistically significant upregulation of the TLR4 (2.7-fold) and TLR5 (3.4-fold) genes was observed in the spleen of *S*. Gallinarum-infected chickens compared to both *S*. Typhimurium-infected animals and uninfected control chickens at 5 days post-infection (Figure 7).

## Discussion

Identification of differences in interaction of *Salmonella* serovars with macrophages from different host species is an important first step in deciphering *Salmonella* pathogenesis and also for possible ways to control infections. Macrophages represent the major phagocyte population that reside underneath the gastrointestinal tract (Smith et al., 2011), and *Salmonella* survival and replication within them have been reported to be essential to the onset of systemic disease in different animals (Haraga et al., 2008; Chappell et al., 2009; Fabrega and Vila, 2013). Therefore, in this study, the interplay between the host generalist *S*. Typhimurium, the host-specific serovar *S*. Gallinarum, and the host-adapted serovar *S*. Dublin and chicken and bovine macrophages was investigated in detail. It is currently unknown to what extent opsonization may affect the interaction with macrophages. However, complement components are present in the intestine *in vivo* (Sina et al., 2018), and further *Salmonella* may experience complement while spreading from one cell to another. Consequently, we included this scenario (opsonized bacteria) in our investigation.

A poor uptake of *S*. Gallinarum was observed in macrophage cell lines of both avian and bovine origin. This is consistent with previous report that *S*. Gallinarum is less invasive than the host generalist serovar *S*. Enteritidis in both chicken and human epithelial cells (Rossignol et al., 2014). Invasion of eukaryotic cell by *Salmonella* is primarily controlled by effectors of the SPI-1 Type Three Secretion System (T3SS) (Haraga et al., 2008; Hayward et al., 2013). The SPI-1 T3SS main effector molecules, *sopA, sopE*, and *sipA*, have single-nucleotide polymorphisms (SNPs) in *S*. Gallinarum strains compared to the host generalist *S*. Enteritidis and *S*. Typhimurium (Thomson et al., 2008; Rossignol et al., 2014; Langridge et al., 2015), and these mutations may explain the poor invasive ability of *S*. Gallinarum in epithelial cells. To what extent this is relevant for uptake in macrophages is unknown. Uptake is believed to be controlled by the macrophage, however, the SPI-1 T3SS is activated upon interaction with macrophages (Pavlova et al., 2011) and mutations in coding genes of T3SS may explain the low uptake in both chicken and bovine macrophages observed in this study. The observation indicates that *S*. Gallinarum is less dependent on uptake in high numbers in macrophages than the other serovars. This correlates well with published observation that SPI-1 is dispensable for *S*. Gallinarum to successfully cause fowl typhoid (Jones et al., 2001).

Once inside HD11 macrophages, *S*. Gallinarum showed poorer net replication than the two other serovars. However, net replication did not differ significantly when the bacteria were opsonized by chicken serum before infections mainly due to increased net replication of *S*. Gallinarum. This indicates that immune components from the serum may interfere with the *S*. Gallinarum survival within chicken macrophages. In contrast, *S*. Dublin and *S*. Typhimurium showed a comparable invading and intracellular net-replication in both chicken and cattle macrophages regardless if the bacteria were opsonized or not. Cattle-derived Bomac were shown to be generally less capable to take up strains of *Salmonella* than HD11 cells but apparently less restrictive on intracellular multiplication and/or less capable of killing intracellular *Salmonella*. Whether this has bearing for the infection in the host, that is, whether cattle macrophages and chicken macrophages use different strategies to limit *Salmonella* infection cannot be concluded from the current study.

*Salmonella* infections induce host cell death as early as 1 h post-infection in a SPI-2 T3SS-dependent manner (Chen et al., 1996; Monack et al., 1996; Li et al., 2009). A significantly higher level of cell death was induced in HD11 cells by *S*. Typhimurium and *S*. Dublin infections compared to *S*. Gallinarum infection. The amount of intracellular viable *Salmonella* decreased to almost the same low level at 4 h post-infection, and it is tempting to speculate that *S*. Gallinarum uses a stealth strategy in its interaction with macrophages of the preferred host to ensure spread to systemic sites. It may be that *S*. Gallinarum expresses surface components that downregulated the apoptotic and pyroptotic pathways in chicken macrophages, and identification of such pathways would be interesting. It may also be that *S*. Gallinarum lacks surface components that are responsible for induction of the signal pathways by the other serovars. The notion that host-specific serovars utilize stealth technique in its preferred host has previously been suggested (Tsolis et al., 2008). In the current study, it was further sustained by observation on Bomac cells, where *S*. Dublin, contrary to its behavior in chicken-derived HD11 cells, showed a low level of cytotoxicity. The broad host range serovar, *S*. Typhimurium, on the other hand, remained highly cytotoxic. A similar result was observed when two more strains from each serovar were tested (Supplementary Figures 2, 3). These results with Bomac were obtained with a very high MOI, and this may have caused unrealistically high numbers of bacteria inside the macrophages. Mouse infection with *S*. Typhimurium resembles host-specific infection in other animals, and indeed, during *in vivo* systemic infection of mice with *S*. Typhimurium, delayed SPI-2-induced macrophage apoptosis was observed (Monack et al., 2001; Grant et al., 2008). This low cytotoxicity has been suggested to facilitate the spread of *Salmonella* in organs by a mechanism involving phagocytosis of infected macrophages by neighboring cells (Monack et al., 2001; Grant et al., 2008; Mastroeni and Grant, 2011). It remains to be clarified in detail whether delayed SPI-2 effect, either due to delayed secretion of effector molecules or low effect on the relevant signal pathways, is also involved in low induction of cytotoxicity by *S*. Gallinarum. Once such an understanding has been obtained, it may be possible to manipulate the responses to the benefit of the host cell.

Activated macrophages produce antimicrobial substances including NO, ROS, and immune mediators to kill intracellular pathogens (Vaughan and Li, 2010; Weiss and Schaible, 2015). At early time points, only very little production of NO was detected for all three serovars, indicating that the synthesis/release may take time during *Salmonella* infection in macrophages *in vitro*. At 24 h post-infection, a significantly higher level of NO production was observed in *S*. Gallinarum-infected cells compared to cells infected with *S*. Typhimurium and *S*. Dublin, indicating that persistent recognition and killing of *S*. Gallinarum probably takes place. This may partially be corroborated by our observation that at 6 h post-infection, a significant downregulation of TLR2, TLR4, and TLR5, which typically recognize the *Salmonella* conserved surface structure, was detected only in *S*. Typhimurium- and *S*. Dublin-infected cells, but not in the *S*. Gallinarum-infected ones. On the other hand, the lower production of NO in *S*. Typhimurium-infected macrophages compared to *S*. Gallinarum-infected cells could be due to the inhibition effect of NO productions by certain molecules from *S*. Typhimurium, and a diminished NO production have previously been observed in *S*. Typhimurium- and *S*. Enteritidis-infected cells compared to cells infected with *S*. Heidelberg and *S*. Kentucky (He et al., 2012). In addition, as we observed a higher level of cell death in *S*. Typhimurium- and *S*. Dublin-infected cells, we suspect that less viable cells may also contribute to the lower NO productions in the *S*. Typhimurium- and *S*. Dublin-infected macrophages.

In line with results of a previous study of chicken and human epithelial cells and chicken macrophages using non-opsonized *Salmonella* (Kaiser et al., 2000; Setta et al., 2012), opsonized *S*. Gallinarum appeared to induce a substantially lower expression of IL1β, CXCLi1, and CXCLi2 compared to opsonized *S*. Typhimurium in HD11 macrophages at 6 h post-infection even though we observed an increased survival for *S*. Gallinarum when this bacterium was opsonized. It seems that opsonization of *S*. Gallinarum mainly influences the survival inside the macrophages and not so much the regulation of inflammation response induced by the uptake of the bacterium. Our study further included *S*. Dublin, which was shown to be more similar to the broad host range *S*. Typhimurium than to the host-specific *S*. Gallinarum. The low induction of inflammation response by *S*. Gallinarum can at least partially be explained by the lack of flagella, since a strain of this serovar, where flagella were present, caused strong induction of IL6 and CXCLi2 expression in primary chicken kidney cells (De Freitas Neto et al., 2013). No doubt other factors than flagella are likely to be important for the differences in host responses to these serovars including the earlier immune response difference. We have recently performed transcriptome analysis of chicken primary macrophages challenge with the same three serovars (Huang et al., 2019) and observed that *S*. Gallinarum induced a much higher number of differential gene expressions in the macrophage than *S*. Typhimurium and *S*. Dublin. Therefore, a comprehensive investigation for the immune modulating factors in *S*. Gallinarum may open new windows for our understanding of the *S*. Gallinarum host specificity and pathogenesis mechanisms. Unexpectedly, and in contrast to the results from the HD11 infections, neither obvious NO production nor differential expression of the immune cytokines, such as IL1β, IL6, IL8, IFN-γ, and IL18 was detected in infected cattle macrophages Bomac (regardless of the infecting serovars) compared to uninfected cells under the same tested conditions, indicating a big difference in the immune response between the chicken and bovine macrophages to *Salmonella* infections.

Intriguingly, in our study, the chicken unique TLR15 was upregulated in the macrophages after infection with all three serovars, indicating that this receptor may be relevant to the general response against *Salmonella* infections in chickens, although its ligand is still not well-defined and recognized. Upregulation of TLR15 in cecum and in heterophils *in vivo* was also observed after *S*. Typhimurium and *S*. Enteritidis infections of chicken *in vivo* (Higgs et al., 2006; Nerren et al., 2009). The role of TLR15 in the chicken defense against *Salmonella* infections needs to be further explored.

Previous research has shown that *Salmonella* host specificity is not determined at the level of infection in the intestine, but that distinct immune responses were triggered at systemic sites between host generalist and host-specific serovars (Jones et al., 2001; Chadfield et al., 2003; Chappell et al., 2009). Therefore, in this study, an examination of expressions of different T-helper cells response genes, pro- and anti-inflammation cytokines, chemokines, and innate immune receptors was performed based on spleen from *S*. Typhimurium- and *S*. Gallinarum-infected chickens. Only innate immune receptor genes TLR4 and TLR5 were seen to differ between *S*. Gallinarum-infected and *S*. Typhimurium infected chickens. It has previously been shown that *S*. Typhimurium infection causes a rapid expression of chemokines and pro-inflammation cytokines, including IL12 and IL18, in the intestinal tissues until 4 days post-infection, but not in the spleen samples (Withanage et al., 2004; Berndt et al., 2007). This is in line with our observation. Intriguingly, compared to host generalist *S*. Typhimurium, *S*. Gallinarum caused distinct regulations of TLR4 and TLR5 expressions, an observation that has also recently been made based on challenge of primary macrophages (Huang et al., 2019), indicating that these receptors play an important role in the outcome of the fowl typhoid infection and that HD11 cells are a suitable model for primary macrophages with respect to expression of these receptor genes.

In conclusion, our study shows that *S*. Gallinarum exhibits lower invasion ability and a relatively lower induction of pro-inflammation responses in macrophages of its preferred host compared to infection with the two other serovars. In contrast to the host generalist *S*. Typhimurium, host-specific *S*. Gallinarum and host-adapted *S*. Dublin showed delayed/lower macrophage death in macrophages of their preferred host species. The finding that both *S*. Gallinarum and *S*. Dublin showed low cytotoxicity in macrophages of their preferred host may be important, as it suggests an infection mode with long-term persistence in the phagocytic intracellular milieu due to low cytotoxicity. Further studies should concentrate on demonstrating this in the animals.

## Data Availability Statement

All datasets generated for this study are included in the article/Supplementary Material.

## Ethics Statement

The animal study was performed with permission to the senior author from the Danish Animal Expectorate (approval no. 2016-15-0201-00870).

## Author Contributions

JO and KH conceived and designed the study. KH performed the experiments. KH, AF, SS, and JO provided the critical advice and contributed to the analysis of data and commenting on the manuscript. All authors read and approved the final manuscript.

### Conflict of Interest

The authors declare that the research was conducted in the absence of any commercial or financial relationships that could be construed as a potential conflict of interest.
