# Supplementary material for: Dynamics and Outcome of Macrophage Interaction Between Salmonella Gallinarum, Salmonella Typhimurium, and Salmonella Dublin and Macrophages From Chicken and Cattle
Source: Front Cell Infect Microbiol. 2020 Jan 10;9:420. doi: 10.3389/fcimb.2019.00420 (PMC6966237; doi:10.3389/fcimb.2019.00420)
Supplement: Supplementary file 1 [file Data_Sheet_1.pdf]

Supplementary Material

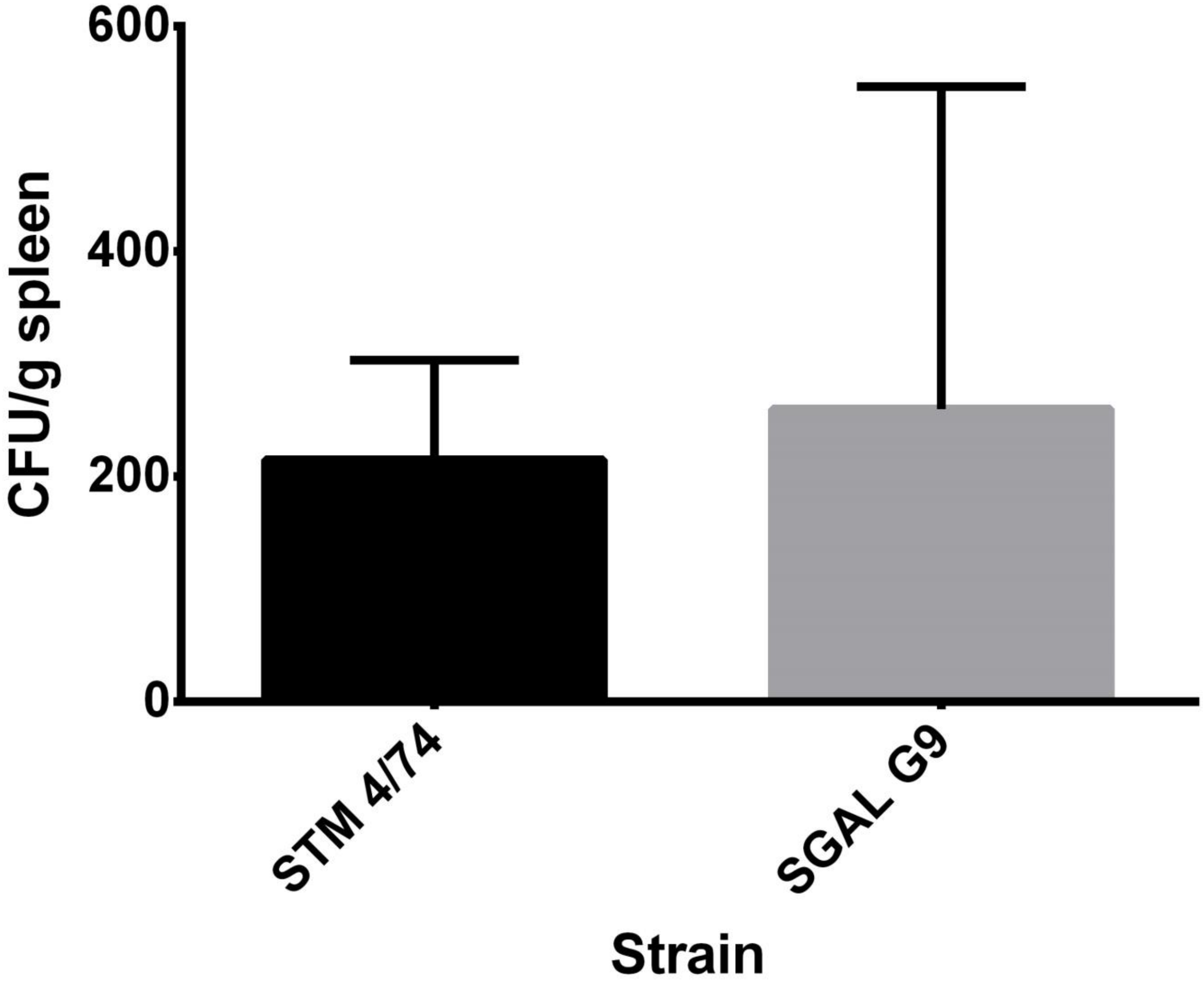

Supplementary Figure S1. Spleen counts of one-week old chicken infected with S. Gallinarum and S. Typhimurium for measurement of induction of immune response genes.

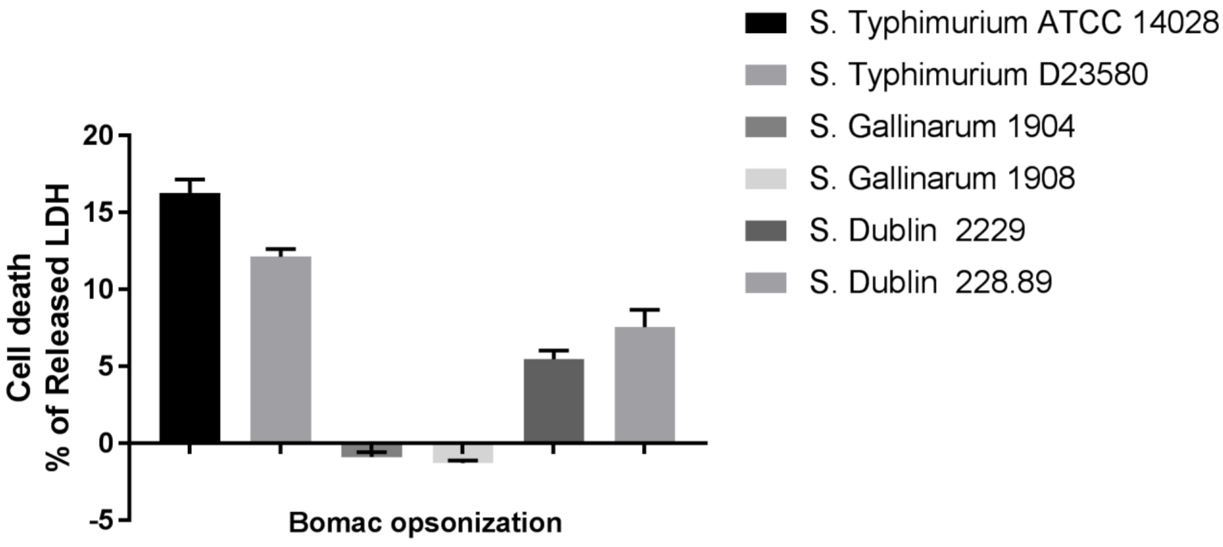

Supplementary Figure S2. Induced chicken macrophage HD11 cell death by *Salmonella* infections. Two more strains from each serovar were used. The HD11 were infected by Salmonella at a multiplicity of 5 for 30 minutes. The triggered cell deaths were determined by the measuring LDH (lactate dehydrogenase) in the cell supernatant at 12 hours post-infection.

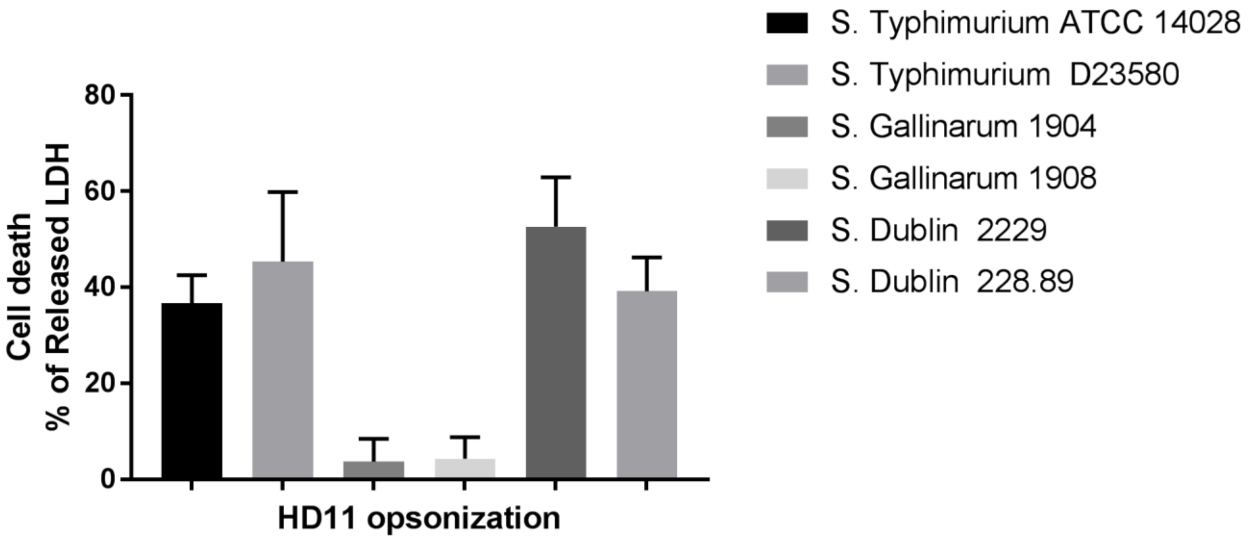

Supplementary Figure S3. Induced bovine macrophage Bomac cell death by *Salmonella* infections. Two more strains from each serovar were used. The Bomac cells were infected by Salmonella at a multiplicity of 100 for 1 hour. The triggered cell deaths were determined by the measuring LDH (lactate dehydrogenase) in the cell supernatant at 12 hours post-infection.
