# Supplementary material for: Dynamics and Outcome of Macrophage Interaction Between Salmonella Gallinarum, Salmonella Typhimurium, and Salmonella Dublin and Macrophages From Chicken and Cattle
Source: Front Cell Infect Microbiol. 2020 Jan 10;9:420. doi: 10.3389/fcimb.2019.00420 (PMC6966237; doi:10.3389/fcimb.2019.00420)
Supplement: Supplementary file 2 [file Table_1.pdf]

Supplementary Material

Supplementary table 1. Primer list for gene expression analysis in HD11 chicken macrophages and spleen by RT-PCR approach.

Primer names	Sequence (5' to 3')	Reference
GAPDH -F	GTCAGCAATGCATCGTGCA	(Berndt et al., 2007)
GAPDH -R	GGCATGGACAGTGGTCATAAGA	
IL-1β -F	GAAGTGCTTCGTGCTGGAGT	(Elsheimer-Matulova et al., 2015)
IL-1β -R	ACTGGCATCTGCCCAGTTC	
IL-6-F	GCGAGAACAGCATGGAGATG	(Jiang et al., 2011)
IL-6-R	GTAGGTCTGAAAGGCGAACAG	
IL-18-F	TCTGGCAGTGGAATGTACTTCG	(Berndt et al., 2007)
IL-18-R	CCATTTTCCCATGCTCTTTCTC	
IFN-γ -F	GCCGCACATCAAACACATATCT	(Elsheimer-Matulova et al., 2015)
IFN-γ -R	TGAGACTGGCTCCTTTTCCTT	
LITAF(TNFα)-F	GCTGTTCTATGACCGCCCAGTT	(Berndt et al., 2007)
LITAF(TNFα)-R	AACAACCAGCTATGCACCCCA	
IL-10-F	CGGGAGCTGAGGGTGAA	(Hong et al., 2006)
IL-10-R	GTGAAGAAGCGGTGACAGC	
CXCLi1-F	CCAGTGCATAGAGACTCATTCCAAA	(Setta et al., 2012)
CXCLi1-R	TGCCATCTTTCAGAGTAGCTATGACT	
CXCLi2-F	GCCCTCCTCCTGGTTTCAG	(Setta et al., 2012)
CXCLi2-R	TGGCACCGCAGCTCATT	
TLR1a-F	ACCCGTTCAAGTGTTCGTG	(Zhou et al., 2013)
TLR1a-R	TTCCGCTCAAGTCTTCTGG	
TLR2b-F	GGTGTTCCTGTTCATCCTCATC	(Zhou et al., 2013)
TLR2b-R	GTTGGAGTCGTTCTCACTGTAGG	
TLR3-F	GCTATTGAGCAAAGTCGAGA	(Zhou et al., 2013)
TLR3-R	ACAGGGGGCACTTTACTATT	
TLR4-F	ATCTTTCAAGGTGCCACATC	(Zhou et al., 2013)
TLR4-R	GGATATGCTTGTTTCCACCA	
TLR5-F	ACTCCCTTCCTTCCCACATCT	(Zhou et al., 2013)
TLR5-R	GTTTGCGAGCCAGTTTCTCTCT	
TLR7-F	TCTGGACTTCTCTAACAACA	(Zhou et al., 2013)
TLR7-R	AATCTCATTCTCATTCATCATCA	
TLR15-F	GGCTGTGGTATGTGAGAATG	(Zhou et al., 2013)
TLR15-R	ATCGTGCTCGCTGTATGA	
TLR21-F	AGTTGTGTCCTGTGCTGAGAGAG	(Zhou et al., 2013)
TLR21-R	AGCAGGTTGTGTTCCACTGTC	

Primers were as described:

1. Berndt, A., Wilhelm, A., Jugert, C., Pieper, J., Sachse, K., and Methner, U. (2007). Chicken cecum immune response to Salmonella enterica serovars of different levels of invasiveness. *Infect Immun* 75**,** 5993-6007.
2. Elsheimer-Matulova, M., Varmuzova, K., Kyrova, K., Havlickova, H., Sisak, F., Rahman, M., and Rychlik, I. (2015). phoP, SPI1, SPI2 and aroA mutants of Salmonella Enteritidis induce a different immune response in chickens. *Vet Res* 46**,** 96.
3. Hong, Y.H., Lillehoj, H.S., Lillehoj, E.P., and Lee, S.H. (2006). Changes in immune-related gene expression and intestinal lymphocyte subpopulations following Eimeria maxima infection of chickens. *Vet Immunol Immunopathol* 114**,** 259-272.
4. Jiang, H., Yang, H., and Kapczynski, D.R. (2011). Chicken interferon alpha pretreatment reduces virus replication of pandemic H1N1 and H5N9 avian influenza viruses in lung cell cultures from different avian species. *Virol J* 8**,** 447.
5. Setta, A., Barrow, P.A., Kaiser, P., and Jones, M.A. (2012). Immune dynamics following infection of avian macrophages and epithelial cells with typhoidal and non-typhoidal Salmonella enterica serovars; bacterial invasion and persistence, nitric oxide and oxygen production, differential host gene expression, NF-kappaB signalling and cell cytotoxicity. *Vet Immunol Immunopathol* 146**,** 212-224.
6. Zhou, Z., Wang, Z., Cao, L., Hu, S., Zhang, Z., Qin, B., Guo, Z., and Nie, K. (2013). Upregulation of chicken TLR4, TLR15 and MyD88 in heterophils and monocyte-derived macrophages stimulated with Eimeria tenella in vitro. *Exp Parasitol* 133**,** 427-433.
