# Supplementary material for: Dynamics and Outcome of Macrophage Interaction Between Salmonella Gallinarum, Salmonella Typhimurium, and Salmonella Dublin and Macrophages From Chicken and Cattle
Source: Front Cell Infect Microbiol. 2020 Jan 10;9:420. doi: 10.3389/fcimb.2019.00420 (PMC6966237; doi:10.3389/fcimb.2019.00420)
Supplement: Supplementary file 3 [file Table_2.pdf]

Supplementary Material

Supplementary table 2. Primer lists for gene expression analysis by RT-PCR approach in Bovine derived macrophages BoMac.

Gene	Primer sequence (5'-3')	Reference
IL1β-F-cattle	GCAACCGTACCTGAACCCA	(Xiao et al., 2016)
IL1β-R-cattle	CACGATGACCGACACCACC	
IL6-F-cattle	GGCGGAGCCTTGCGTTAT	(Xiao et al., 2016)
IL6-R-cattle	AACTGCTGTGCTTGCTTCAT	
IL10-F-cattle	TATCCACTTGCCAACCAGCC	(Xiao et al., 2016)
IL10-R-cattle	TCTTCAGCTTCTCCCCCAGT	
IL12-F-cattle	TTCGCAGCCTCCTCCTCATA	(Xiao et al., 2016)
IL12-R-cattle	GTTTTGGGAGTAGTCGAGGCAG	
TNFa-F-cattle	CCAGGCAACTTGCTCTCTCT	(Xiao et al., 2016)
TNFa-R-cattle	GCTGAGGCACAAGCAACTTC	
GAPDH-F-cattle	GATGGTGAAGGTCGGAGTGAAC	(Xiao et al., 2016)
GAPDH-R-cattle	GTCATTGATGGCGACGATGT	
Cattle-TGFβ-F	CATCTGGAGCCTGGATACACAGT	(Palomares et al., 2014)
Cattle-TGFβ-R	GAAGCGCCCGGGTTGT	
Cattle-TLR2-F	TGCGTTGGTTTGGATAGTGA	(Silva et al., 2012)
Cattle-TLR2-R	AGGATGATGACAGCCCAGAC	
Cattle-TLR4-F	GACCCTTGCGTACAGGTTGT	(Silva et al., 2012)
Cattle-TLR4-R	GGTCCAGCATCTTGGTTGAT	
Cattle-TLR5-F	TCAATGGGAGCCAGATTTTC	(Silva et al., 2012)
Cattle-TLR5-R	CCCAGCAAAGGTGTGGTAGT	

Primer sequences were obtained from the following studies:

1. Palomares, R.A., Brock, K.V., and Walz, P.H. (2014) Differential expression of pro-inflammatory and anti-inflammatory cytokines during experimental infection with low or high virulence bovine viral diarrhea virus in beef calves. *Vet Immunol Immunopathol* **157**: 149-154.
2. Silva, A.P., Costa, E.A., Macedo, A.A., Martins Tda, M., Borges, A.M., Paixao, T.A., and Santos, R.L. (2012) Transcription of pattern recognition receptors and abortive agents induced chemokines in the bovine pregnant uterus. *Vet Immunol Immunopathol* **145**: 248-256.
3. Xiao, J., Xie, R., Li, Q., Chen, W., and Zhang, Y. (2016) Generation and characterization of bovine bone marrow-derived macrophage cell line. *Cell Biol Int* **40**: 603-608.
